# Planning and development of an antimicrobial stewardship program in penitentiary facilities: strategies to optimize therapeutic prescribing and reduce the incidence of antibiotic resistance

**DOI:** 10.3389/fpubh.2023.1233522

**Published:** 2023-10-25

**Authors:** Ludovica Mazzoleni, Andrea Zovi, Cinzia D'Angelo, Cecilia Borsino, Nicola Cocco, Raffella Carla Lombardo, Roberto Ranieri

**Affiliations:** ^1^Pharmacy, Santi Paolo e Carlo Hospital, Milan, Italy; ^2^Department of Pharmaceutics, ATS Metropolitan City of Milan, Milan, Italy; ^3^Penitentiary Infectious Diseases Unit, Santi Paolo e Carlo Hospital, Milan, Italy

**Keywords:** antimicrobial stewardship, antibiotic-resistant bacteria, bacterial infections, inmates, correctional facilities, prisons

## Abstract

**Introduction:**

In correctional facilities, due to the high incidence of bacterial infections, antibiotics are widely prescribed. As a result, it may occur a massive and improper use of antibiotics, which promotes the development of antibiotic-resistant bacteria. However, in literature, specific experiences, interventions or guidelines aimed to optimize their prescription within prisons are sporadic.

**Objectives:**

In an Italian hospital where belong patients from four penitentiary institutions, a multidisciplinary team has implemented an antimicrobial stewardship project. The aim of the project was to reduce the incidence of antibiotic resistance in penitentiary institutions by optimizing and rationalizing antibiotic prescribing.

**Methods:**

Following the analysis of microbiological prevalence and antibiotic consumption data within correctional facilities, the Antimicrobial Stewardship Team developed operational tools to support prison healthcare staff to manage properly antibiotic therapies.

**Results:**

The analysis showed a gradual increase in antibiotic resistance: in 2021 the prevalence of resistant microorganisms was 1.75%, four times higher than in 2019. In contrast, between 2019 and 2021, antibiotic consumption decreased by 24%. Based on consumption data, pharmacy has drafted an antibiotic formulary for correctional facilities, supplemented with guidelines and data sheets, and also developed a prescription form for critical antibiotics.

**Conclusion:**

Results showed an increasing incidence of antibiotic resistance within prisons, highlighting the need to establish a dedicated antimicrobial stewardship program. This project may impact positively not only on prisoners, but also for the entire community, as prisons can be considered as places of health education and promotion.

## Introduction

The significant incidence of bacterial infections in prison facilities is currently one of the main critical issues plaguing prison health care. HIV infection and viral hepatitis are the most common infectious diseases affecting people living in prison (PLP) (1), although local or systemic bacterial infections are also recurrent, such as skin and soft tissue infections, respiratory tract infections, urinary tract infections, sexually transmitted infections (STIs), tuberculosis, otolaryngological and dental infections (2, 3). Moreover, a massive and improper use of antibiotic drugs promotes the development of resistant or multidrug-resistant bacteria. Therefore, clinical management of infections is increasing difficult and the availability of effective agents is reduced (4, 5). Overall, PLP have a crucial role in the spread of antibiotic resistance. In fact, imprisonment associated to a compromised health status may expose PLP to an increased risk of contracting infections, which can be followed by an inappropriate use of antibiotics. The consequence is a wide spread of resistant microorganisms, both within prisons and then within the community at large, due to the rapid turnover of some inmates who often move from one correctional facility to a second prison (6). Currently, in the scientific literature there is little evidence compared to the trend of this phenomenon in the penitentiary setting and the development of best-practice tools useful to make in practice effective actions to prevent antimicrobial resistance (AMR) in prisons. In particular, in penitentiary institutions belonging to the Italian region where this study took place, no antimicrobial stewardship practice has ever been implemented to address the problem of AMR in the real life setting. According to WHO, antimicrobial resistance will be the leading cause of human death by 2050. Consequently, even in a context such as the prison setting, it is crucial to implement and foster concrete actions which may address this phenomenon between now and the coming decades.

## Objectives

In order to counteract the spread of this phenomenon, a multidisciplinary team from an Italian hospital to which the four Milan penitentiary institutions belong, has implemented an antimicrobial stewardship project. The project aimed to provide operational tools to optimize prescription, rationalize the use of antibiotics and promote the safety of drug therapies by reducing the risk of prescription mistakes and antibiotic-related toxicity in prison, with the aim of decreasing the incidence of antibiotic resistance in this particular setting. Furthermore, this work aimed to analyze the microbiological prevalence and local consumption of antibiotics in Milan penitentiary setting between 2019 and 2021, describing all the interventions implemented for the development of a prison-specific antimicrobial stewardship program.

## Methods

### Setting and study design

The project has been developed through two steps and it has been addressed to the four penitentiary institutions in Milan (one remand house, San Vittore, two long-sentenced prisons, Opera and Bollate, and Beccaria, a juvenile prison). A multicenter retrospective epidemiological survey of the four penitentiary institutions has been carried out, to analyze microbiological prevalence and local antibiotic consumption data. Sample included prison population at Milan penitentiary facilities between 2019 (3665 PLP), 2020 (3346 PLP), and 2021 (3316 PLP). Data have been compared over this period, to assess trends in antibiotic consumption and in bacterial resistance. Based on results of the previous analysis, an antimicrobial stewardship program has been implemented. The program involved several health care professionals and provided for the development of operational tools aimed to support antibiotics prescription. Actions undertaken were addressed to a sample consisting of the population detained in Milan prisons since the beginning of the project. In particular, sample included 3540 adults, of which 192 women, 40 young male juveniles and 5 mothers with their 5 children. 40.5% of PLP were foreigners (7). The average age was 41 years, with a wide range of ages, due to the presence of juvenile prison and children up to 6 years hosted at the attenuated custodial institution (ICAM) for mother living in prison. The condition of incarceration at one of the Milan correctional institutions in the years under investigation was the inclusion criterion adopted. All PLPs who were no longer incarcerated by 2019, due to transfers to other prisons or release from prison, have been excluded.

### Collection of microbiological prevalence data

The microbiology unit shared microbiological prevalence data, which have been obtained through the collection and analysis of microbiological sample reports from correctional facilities, referring to the years 2019, 2020, and 2021.

### Collection of antibiotic consumption data

Antibiotic consumption data have been provided by the hospital pharmacy. In particular, the analysis focused on data for the years 2019, 2020, and 2021, which have been extracted from the hospital’s management software. The data have been entered into a database containing the list of antibiotic drugs used in prisons, classified by Anatomical Therapeutic Chemical classification system (ATC) and marketing authorization code (MA code), dosage and pharmaceutical form. In addition, for each drug the database reported the Defined Daily Dose (DDD) value and the annual consumption, defined as the amount of antibiotic, expressed by the number of dosage units, used in penitentiary facilities in the period analyzed. According to the National Plan to Face Antimicrobial Resistance, approved in Italy in 2022, which assesses trends in territorial antibiotic consumption in DDD/1000 population per day (8), the following formula has been applied, to evaluate the consumption trend: DDD value of each antibiotic × the amount of antibiotic used × 1000/number of inmates. As a result, it has been obtained a unique indicator which provided a real and standardized representation of antibiotic consumption, compared with penitentiary population’ size. Values have been summed by grouping the different active agents by ATC, in order to evaluate the overall consumption, according to the different classes of antibiotics.

### Project development

The project included the development of an Antimicrobial Stewardship Team, including the following professional figures: infectious diseases specialist, pharmacist, nurse, microbiologist and health management physician. Team members attended a series of multidisciplinary meetings to analyze local epidemiological data, to identify the main purposes and working methods, and to periodically assess the advancement of the project.

After the collection and the review of microbiological prevalence and antibiotic consumption data, the Antimicrobial Stewardship Team addressed the following actions:

- based on antibiotic consumption data, the identification of active agents used in prisons, in order to draft a specific formulary for antibiotic therapies;
- the development of data sheets for each antibiotic included in the formulary, to guide physicians in prescribing and supporting nurses in the proper management of antibiotic therapies;
- the identification of critical antibiotics, which require approval by an infectious diseases specialist before their administration, and the development of a specific request form for their prescription;
- the drawing up of guidelines for the treatment of the most common infections in penitentiary institutions.

## Results

### Analysis of microbiological prevalence data

In 2019, 2020, and 2021 the microbiology unit analyzed a total of 742 microbiological samples coming from Milan prisons: 251 samples have been analyzed in 2019, 232 and 229 in 2020 and 2021, respectively. Most of them were respiratory or urinary samples. The results showed the presence of 7 antibiotic-resistant bacteria. In 2019, only a strain of *Proteus mirabilis* with a multiresistance profile to penicillins, cephalosporins, ciprofloxacin, colistin, fosfomycin, tigecycline, and to the trimethoprim-sulfamethoxazole association, has been identified. The bacteria infected a prisoner at the San Vittore penitentiary. In 2020, two microorganisms have been isolated in two people living in San Vittore prison: a β-lactamase-producing *Enterobacter hormaechei* resistant to penicillins and cephalosporins, and an extended-spectrum β-lactamase (ESBL)-producing *Escherichia coli* strain.

In 2021, the following microorganisms have been isolated in four PLP detained in Bollate and San Vittore prisons:

- an ESBL-producing *Escherichia coli* resistant to penicillins, cephalosporins, fluoroquinolones, and to the trimethoprim-sulfamethoxazole association;
- an ESBL-producing *Klebsiella pneumoniae*;
- a multiresistant *Morganella morganii* strain, specifically resistant to amikacin, penicillins, cephalosporins, ciprofloxacin, ertapenem and fosfomycin;
- an ESBL-producing *Escherichia coli* strain.

All microorganisms have been detected in PLP’s urine and processed by urinoculture.

Antibiotic-resistant bacteria accounted for 0.94% of all microbiological isolates coming from Milan prisons, from 2019 to 2021. The analysis showed a progressive increase in antibiotic resistance: in 2019 and 2020 the prevalence was 0.39 and 0.86%, respectively, whereas in 2021 there was an increase of the prevalence up to 1.75% (Table 1).

**Table 1:** Trend of antibiotic resistance in Milan penitentiary facilities in 2019, 2020, and 2021.

	2019	2020	2021
Analyzed samples	251	232	229
Multidrug-resistant bacteria isolated	1	2	4
Prevalence	0.39%	0.86%	1.75%

### Analysis of antibiotic consumption data

**Figure 1:**
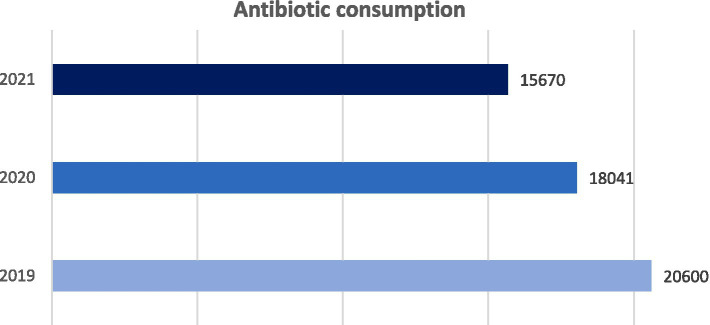
Trend of total antibiotic consumption in Milan correctional facilities from 2019 to 2021. Consumption: DDD × amount of antibiotic used × 1000/number of inmates in the reference year.

The analysis conducted by the hospital pharmacy showed a gradual decrease in antibiotic consumption within correctional facilities. In 2019, a total antibiotic consumption of 20600 DDD has been found. In 2020, this figure decreased by 12%, going to 18041 DDD. In 2021, the total consumption of antibiotics decreased further, settling around the value of 15670 DDD, 13% less than the previous year (Figure 1 and Table 2). Consistent with local and national data, this phenomenon was probably related to the lower incidence of infections among inmates detected during the COVID-19 pandemic (9).

**Table 2:** Number of adult people living in Milan prisons in 2019, 2020, and 2021.

**Size of penitentiary population**			
**Prison**	**2019**	**2020**	**2021**
Bollate	1347	1241	1209
San Vittore	984	921	930
Opera	1334	1184	1177
Total	3665	3346	3316

Analyzing the consumption trend of the most widely used systemic antibiotics in Milan prisons, in 2021, compared with 2019, a lower use of the associations of penicillins and β-lactamase inhibitors has been observed. In particular, the consumption of amoxicillin-clavulanic acid tablets at the dosage of 875 mg + 125 mg decreased by 21%, going from 9914 DDD in 2019 to 7791 DDD in 2021. However, amoxicillin-clavulanic acid was still the most widely used antibiotic. Macrolides, such as clarithromycin and azithromycin, were also widely prescribed. In this case, the analysis showed a 23% reduction in consumption between 2019 and 2021, although there has been a sudden increase in 2020, especially for azithromycin (from 1246 DDD in 2019 to 2593 DDD in 2020, and then decreased to 1416 DDD in 2021). This significant increase in 2020 was likely due to the fact that azithromycin was initially recommended for the treatment of COVID-19 in non-hospitalized patients (8). Consumption of systemic fluoroquinolones in prisons amounted to 1772 DDD in 2021: it is noteworthy that compared to 2019, in 2020 it dropped by 24%, and in 2021 it decreased further by 11%. The most widely used fluoroquinolone agent was levofloxacin, whose consumption in 2019, 2020, and 2021 was, respectively, of 1853 DDD, 1219 DDD, and 1043 DDD. Among the broad-spectrum penicillins, the most widely prescribed antibiotic was amoxicillin 1000 mg. Its consumption was 2554 DDD in 2019, and it dropped to 1465 DDD in 2021, with an overall reduction of 43%. In contrast, the consumption of tetracyclines increased by 25%. In particular, the consumption of doxycycline increased from 655 DDD in 2019 to 826 DDD in 2021, with a slight decrease of 7% in 2020, compared to the previous year. Table 3 illustrates the consumption trends of all the classes of antibiotics used within the Milan prisons, between 2019 and 2021.

**Table 3:** Trends in antibiotic consumption grouped by ATC in Milan prisons between 2019 and 2021.

**DDD/1000 PLP**	**Year**		
**ATC description**	**2019**	**2020**	**2021**
Association of penicillins and beta lactamase inhibitors	9933	8367	7803
Macrolides	3218	3949	2483
Fluoroquinolones	2608	1987	1772
Broad-spectrum penicillins	2570	1673	1473
Tetracyclines	662	609	826
Third-generation cephalosporins	222	238	181
Associations of sulfonamides and trimethoprim	191	146	144
Nitrofurantoin	19	9	3
Other antibacterial agents	15	11	30
Aminoglycosides	5	7	6
Carbapenems	7	4	4
Glycopeptides	6	1	4
Beta lactamase-sensitive penicillins	4	3	2
Lincosamides			2
First-generation cephalosporins			1
Total	20600	18041	15670

### Implemented measures to promote appropriate prescribing and proper use of antibiotics

Based on these results, the pharmacy developed both a formulary of antibiotic therapies for correctional facilities and a request form aimed to address highly critical antibiotics’ prescriptions. The antibiotic formulary was aimed at providing a useful tool for clinicians in the empirical choice of antibiotic therapy to deal with more critical infectious diseases which may occur in correctional institutions. All antibiotics used in Milan prisons have been listed and divided by pharmacological class and ATC. As part of the antimicrobial stewardship process, the use of this formulary requires specific training of prison medical staff, which is based on two key points: the need to collect biological samples for etiological identification before starting any antibiotic therapy, and the timely adaptation of the empirical therapy after the microbiological results. The prescription form aimed at ensuring greater control over the prescribing and use of antibiotics, through the direct involvement of the clinician and the pharmacist. It has been designed for prescribing antibiotics which do not belong to the first line of treatment, which have specific prescribing restrictions or that require special precautions in management and administration. Before their use, these agents required approval by an infectious diseases specialist. Some instances are glycopeptides such as vancomycin and teicoplanin, carbapenems, daptomycin, linezolid, colistin, tigecycline, intravenous ampicillin, dalbavancin and latest generation cephalosporins. The form included information about the requesting unit and the prescribing doctor, patient’s data (including age and body weight), the required antibiotic, its dosage, duration of treatment, whether it was prescribed as monotherapy or in combination with other medicines, its clinical indication, specifying whether the treatment was empiric or targeted and the name of the isolated microorganism, any previous antibiotic therapies. Eventually, the last section of the form required specifying whether the prescribed medication had been approved by an infectious diseases specialist. After verifying the request’s appropriateness, according to antimicrobial prescribing criteria, the pharmacist can authorize it. The hospital pharmacy also prepared data sheets for each antibiotic included in the formulary, to provide support to healthcare professionals in prescribing and managing antibiotic therapies. Each data sheet contained information about pharmacokinetics, dosage, interactions, major side effects, methods of preparation and storage. The formulary has been supplemented with specific guidelines for the treatment of the most recurrent infections in prisons: skin and soft tissue infections, STIs, urinary tract infections, otorhinolaryngological infections and pulmonary infections. The guidelines have been developed by the pharmacy, in cooperation with the infectious diseases specialists working in prisons. They referred to both community and nosocomial infections, as PLP could develop infections before and/or during the incarceration. Moreover, they could also contract care-related infections during any hospitalization and, once discharged, they could continue their care within correctional facilities. They focused on antibiotic therapies, distinguishing between empiric therapies and following treatment lines, and indicating which active agents must be chosen once detected antibiotic resistance, specifying any particular contraindication or recommendation for allergic patients or pregnant women who live in prison.

## Discussion

From a social perspective, prison communities are largely represented by individuals with great difficulties, such as homeless or people with mental disorders or drug addictions (10, 11). These people are at increased risk of infection (12). In addition, several risk factors related to detention setting, such as overcrowding, poor hygienic conditions, lack of effective preventive measures, promiscuity and poorly ventilated environments, may promote occurrence and transmission of infections (13, 14). Reduced availability of diagnostic tests, difficult access to microbiological tests and high mobility of PLP can delay diagnosis of infections, complicating identifications of infectious outbreaks, to stop transmission and to eradicate the disease (2). This increases risk of contagion and spread of new infections (2, 15). In a context of poor and difficult infection management, often there is a massive and improper use of antimicrobial drugs within correctional facilities, in particular of antibiotics, which causes the onset of antibiotic resistance. AMR commonly occurs in individuals with a compromised immune system, for example those with AIDS, cancer, or who have received an organ transplant (5). Other factors that may contribute to its development are close contact and promiscuity with strangers, especially if not vaccinated, drug abuse and smoking (5, 6, 14). In the community this phenomenon affects both men and women, while in prison mainly the male population, which represents the majority. In literature there are few data describing AMR and the inappropriate use of antibiotics within correctional institutions. Due to the lack of data in this context, it is crucial to compare results of this study with other findings, although this project could be innovative for the scientific community. However, limited evidence in literature is consistent with our results. In fact, a study which analyzed antibiotics prescribed for the treatment of acute upper respiratory tract infections (URTIs) and dental infections in Italian prisons showed that overprescription of antibiotics is widespread in the prison population, accounting for about 69.4% of the prescriptions (16). Another study found that antibiotics agents are prescribed in 67% of cases of respiratory infections diagnosed in prison settings, still underestimating the large use of these drugs and highlighting a low utilization of diagnostic tests, in particular microbiological tests (17). There may be several factors behind the overprescribing of antibiotics in prison facilities. For instance, most of the diagnoses of infections in prison facilities are based on clinical signs and symptoms, whereas microbiological examinations and diagnostic tests are less used. As a result, empirical prescription of broad-spectrum antibiotics is preferred over targeted therapies (16). Often prison internists prescribe broad-spectrum oral antibiotics, without requiring specialist or pharmacist advice. As a result, the incidence of inappropriate prescribing may increase the use of inappropriate antibiotics. Another reason could be related to a higher perceived risk of development of serious bacterial infections by PLP, as well as to a potential requirement for increased follow-up when antimicrobials are not prescribed. Furthermore, it has been reported that not prescribing antibiotics may be perceived by prison patients as a “non-cure,” believing that these drugs are harmless (16, 18). In addition, the existence of an underlying chronic clinical condition in PLP is a further potential cause of bacterial resistance. In fact, patients with comorbidities are at high risk of developing antibiotic resistance, due to their vulnerability to infections and to the relative frequent exposure to antibiotic treatments (16, 19). Antimicrobial stewardship has proven to be an effective tool to rationalize antibiotic consumption, improving prescribing appropriateness (20, 21). Several actions have been implemented in hospitals and in different care settings (22). However, despite of the presence of a high infectious risk, effective measures in Italian prisons are lacking (23). Therefore, a dedicated antimicrobial stewardship project for Milan penitentiary facilities has been implemented, with a specific focus on antibacterial drugs. The results of this study showed a relatively low incidence of infections caused by multidrug-resistant microorganisms, as well as a gradual reduction in antibiotic consumption from 2019 to 2021. Nevertheless, it is noteworthy that prevalence of multidrug-resistant superbacteria infections in the three-year period analyzed followed an increasing trend, doubling from 2019 to 2020, and then again from 2020 to 2021. However, this study has some limitations. In fact, collected data may have been strongly affected by the COVID-19 pandemic, which led to a lower incidence of infections within prisons, probably due to the adoption of preventive measures such as the use of personal protective equipment and the implementation of hygiene and sanitation measures (24, 25). In addition, the high turnover of PLP resulting from new entries, releases or transfers to other prisons did not guarantee a defined and constant sample throughout the duration of the project. The project is still under development. Training meetings will be organized with medical staff, in order to promote the use of new prescription form and therapeutic formulary, as well as to educate and to sensitize prison staff on the proper management of antibiotic therapies. Microbiological diagnostic services will be improved to incentivize the prescribing of targeted antibiotic therapies based on the antibiogram, thereby limiting the prescription of broad-spectrum empirical therapies. Eventually, some indicators related to antibiotic consumption and microbiological prevalence will be continuously monitored over time, with the aim to assess the effectiveness of the project.

## Conclusion

The results of this study show that in Milan prisons, in recent years there has been a high number of empirical prescriptions, as well as a massive use of broad-spectrum antibiotics. This led to an increasing trend in the development of antibiotic-resistant bacteria. Therefore, it is crucial to establish an antibiotic stewardship program within correctional institutions, which provides supportive operational tools and defines a shared pathway aimed at improving the prescriptive appropriateness of antimicrobial therapies. In addition, the implementation of an antimicrobial stewardship project could be a significant initiative not only to improve PLP health conditions, but also to positively impact on the whole community. In fact, detention represents a health risk factor due to several determinants, such as social and cultural marginalization of penitentiary population, overcrowding, poor hygienic conditions and high turnover. However, prison often represents a contact point with the National Health System and an environment to prevent, diagnose, and treat infectious diseases.

## Data availability statement

The original contributions presented in the study are included in the article/supplementary files. Further inquiries can be directed to the corresponding author.

## Author contributions

LM, CD’A, and RR: conceptualization. LM, AZ, and CD’A: methodology. LM, CB, NC, and CD’A: investigation. LM, AZ, and RR: writing – original draft preparation. CD’A, CB, NC, and RL: writing – review and editing. CD’A, CB, NC, RL, and RR: visualization. All authors contributed to the article and approved the submitted version.
